# Effects of Dietary Fiber Compounds on Characteristic Human Flora and Metabolites Mediated by the Longevity Dietary Pattern Analyzed by In Vitro Fermentation

**DOI:** 10.3390/nu14235037

**Published:** 2022-11-26

**Authors:** Fengcui Shi, Fan Zhou, Xiaohua Zheng, Jingwen Lv, Xiaohan Yu, Yang Zhou, Quanyang Li

**Affiliations:** 1 College of Light Industry and Food Engineering, Guangxi University, Nanning 530004, China; 2 Wangdingdi Hospital, Tianjin Nankai District, Tianjin 300191, China

**Keywords:** longevity dietary pattern, dietary fiber compound, in vitro fermentation, characteristic human flora, metabolites

## Abstract

The purpose of this study was to investigate the effects of different dietary fiber compounds (DFCs) on characteristic human flora and their metabolites mediated by the longevity dietary pattern analyzed by in vitro fermentation. The results show that DFC1 (cereal fiber) increased the level of *Lactobacillus* (*p* < 0.05), DFC2 (fruit and vegetable and cereal fiber) promoted the growth of *Lactobacillus* and *Bifidobacterium* more significantly than DFC3 (fruit and vegetable fiber) (*p* < 0.01), and all three DFCs decreased the level of *Escherichia coli* (*p* < 0.05). The metabolomic analysis showed that there was variability in the metabolites and the metabolic pathways of different DFCs. The redundancy analysis revealed that the fiber content was positively correlated with *Lactobacillus*, *Bifidobacterium*, *Bacteroides*, acetic acid, butyric acid, propionic acid, lactic acid, and betaine, and negatively correlated with *Escherichia coli*, succinic acid, alanine, choline, aspartic acid, and α-glucose. Overall, this study found that different DFCs have different positive correlations on characteristic human flora and metabolites, and DFC2 is more favorable to the proliferation of the intestinal beneficial genera *Lactobacillus* and *Bifidobacterium* after in vitro fermentation, having a probiotic role in glucose, amino acid, and lipid metabolisms. This study may provide a theoretical reference for the search of optimal dietary fiber combination strategies mediated by longevity dietary pattern.

## 1. Introduction

It is generally agreed that dietary fiber (DF) has an important role in human health, and it mainly exerts its function by regulating the structural composition of gut microbes in humans [1,2]. Prebiotics consisting of a single dietary fiber polysaccharide or several random combinations of polysaccharide components have been developed [3], especially oligofructose, oligosaccharide, and oligogalactose, which have been marketed in large quantities. Recent studies have found that different dietary fiber polysaccharides have different effects on the regulation of gut microbes [4], and the positive regulation of gut microbes by DF with a single component is unknown or uncertain, and its effect is limited, sometimes even failing to exert health-promoting effects. Therefore, it is necessary to explore the compounding of DF and ensure that the compound is more beneficial to human health. This is a very complex issue if we consider the different compositions and content of dietary fiber, and at the same time, a reasonable DF nutrition strategy has great scientific value. A team investigated the dietary characteristics of the longevity population in Guangxi, and the traditional diet in the Bama region was mainly based on plant-based foods, such as coarse grains, vegetables, and fruits, with a relatively high dietary fiber intake [5,6]. Based on the results of their preliminary research, combined with the recommended dietary nutrient intake of Chinese residents, the team created a dietary pattern that reflected the dietary characteristics of the longevity population and found that the dietary pattern has good anti-aging effects and development potential [7,8]. To this end, this study is conducted to investigate the effects of different DFCs on characteristic human flora and their metabolites under the longevity dietary pattern through in vitro experiments, based on the concept of “asking the centenarians about their dietary patterns and seeking strategies for dietary fiber combination”.

In terms of the role of DF in maintaining intestinal health, Ge [9] used an in vitro fermentation model to investigate the effect of insoluble dietary fiber from bamboo on human gut microbes and found an increase in the total short-chain fatty acid production, an increase in the relative abundance of *Bacteroides*, and a decrease in the ratio of *Firmicutes* to *Bacteroides* (F/B). Relative to single-component DF, one study found that soluble dextran produced greater changes in gut microbes than pectin polymers after in vitro fermentation [10]. Ren [11] found that centenarian-derived *Lactobacillus* LTL1361 combined with DFC attenuated age-related cognitive impairment and protected brain and intestinal function. Most of these studies have explored the effects on in vitro gut health in terms of a single DF or several complex DFs, while the modulation of gut microbes from different dietary fiber compounds in a specific dietary pattern has not been reported.

In vitro colon simulators offer the advantage of supporting dynamic sampling for characterizing the metabolic status of DF in the human body, and they face fewer ethical issues compared to in vivo studies [12]. The fermentation of DF in the colon was simulated by in vitro fermentation methods, making it easier to adopt standardized fermentation conditions, determine fermentation times, measure the number of end products, and lower costs [13,14].

In summary, this study investigates the effects of DFCs on characteristic human flora and metabolites after in vitro fermentation from different types of DFCs (cereal, fruit and vegetable and cereal complex, and fruit and vegetable), mediated by the longevity dietary pattern, in order to better provide some theoretical support for human health enhancement through the optimization of dietary fiber.

## 2. Materials and Methods

### 2.1. Preparation of DFCs

In the longevity dietary pattern, cereal fiber and fruit and vegetable fiber are the main dietary fiber types with a relatively high fiber content. In this study, cereal fiber and fruit and vegetable fiber were mixed in proportion to form three equal dietary fiber compounds, namely, DFC1 (cereal fiber), DFC2 (fruit and vegetable and cereal fiber), and DFC3 (fruit and vegetable fiber), whose dietary structure and daily food intake ratios were based on the Guangxi longevity dietary pattern constructed by a previous team [5,6,7,8]. Additionally, the no dietary fiber (NDF) group (i.e., the composition is the same as that of the DFC group except that no dietary fiber compound is added) was chosen as the experimental control group. Then, equal amounts of the extracted dietary fiber compounds were taken for subsequent analysis.

The DFC1 extraction conditions were as follows: to the liquid-to-material ratio of 15 mL/g, add 0.5% α-amylase, adjust pH to 10.0, adjust ultrasonic power 240 W to assist enzymatic digestion for 20 min, place in a water bath at 70 °C, extract within 90 min, inactivate enzyme at 100 °C for 10 min, centrifuge at 8000 r/min for 15 min, and freeze-dry to obtain insoluble dietary fiber. DFC2 was extracted under the following conditions: to the liquid-to-material ratio of 15 mL/g, add 0.3% α-amylase, adjust the extraction temperature of 86 °C, adjust pH to 5.0, adjust ultrasonic power to 242 W to assist enzymatic digestion for 20 min, place in a water bath at 86 °C for 90 min, and extract at 100 °C. After the inactivation of the enzyme in a water bath for 10 min, centrifugation at 8000 r/min for 15 min, and freeze-drying to obtain insoluble dietary fiber, the supernatant was mixed with 95% ethanol for alcohol deposition, centrifuged, and freeze-dried to obtain soluble dietary fiber. DFC3 was extracted under the following conditions: place the liquid-to-material ratio of 20 mL/g in a water bath at 80 °C for 90 min, centrifuge at 8000 r/min for 15 min, freeze-dry to obtain insoluble dietary fiber, and add the supernatant to 95% ethanol for alcohol precipitation, centrifuge, and freeze-dry to obtain soluble dietary fiber.

### 2.2. Determination of Nutrient and Fiber Contents

The water content in the samples was determined according to GB5009.3-2016 [15]; the ash content was determined according to GB5009.4-2016 [16]; the protein content was determined according to GB5009.5-2016 [17]; the fat content was determined according to GB5009.6-2016 [18]; the pectin, cellulose, hemicellulose, and lignin were determined according to the modified procedure of Ma [19] and Capek [20]. The sample (y) was mixed with a 0.25% ammonium oxalate solution in a ratio of 1:10 (M/V) and stirred continuously in a water bath at 90 °C for 2 h. The supernatant was collected by centrifugation and the above operation was repeated twice; the supernatant was mixed and dialyzed in a dialysis bag (retained molecular weight: 7000 U) until the conductivity was the same as that of deionized water, and the retained solution was collected and freeze-dried to obtain pectin (A1). The above filter residue was washed twice with 80% ethanol and three times with distilled water and then lyophilized. The lyophilized residue was mixed with a potassium hydroxide solution containing 0.1% sodium borohydride (4 mol/L) at a material–liquid ratio of 1:30 (M/V) at room temperature, stirred, and extracted for 24 h. After filtration, the filtrate was neutralized with an acetic acid solution (4 mol/L), dialyzed with a dialysis bag until the conductivity was the same as that of deionized water, and the intercepted solution was freeze-dried to obtain hemicellulose (A2). The filtered residue was washed and lyophilized to obtain the sample A3; concentrated sulfuric acid solution (72%, M/M) was added in the ratio of 1:1 (M/V) to A3 and extracted for 24 h at 4 °C in the refrigerator, filtered in turn, washed, and dried to obtain the sample A4, whose ash mass was A5.

(1) $$\mathrm{Cellulose}\;\mathrm{content}\;(\mathrm{on}\;a\;\mathrm{dry}\;\mathrm{basis},\%)=\frac{A_{3}(g)-A_{4}(g)-A_{5}(g)}{Y(g)}\times 100$$

(2) $$\mathrm{Lignin}\;\mathrm{content}\;(\mathrm{on}\;a\;\mathrm{dry}\;\mathrm{basis},\%)=\frac{A_{4}(g)-A_{5}(g)}{Y(g)}\times 100$$

### 2.3. In Vitro Fermentation Experiment

#### 2.3.1. Sample Preparation

Four volunteers (two males and two females) in the age range of 65–75 years were recruited to collect fresh human fecal samples. The volunteers were required to be in good health, non-smokers, non-drinkers, free of intestinal diseases (such as diabetes, kidney disease, and liver disease), and not having taken antibiotics within the previous 3 months. The volunteers were informed of the purpose of this study before sampling and their approval was obtained in full accordance with ethical procedures. A total of 20 g of fresh fecal samples from four volunteers was taken (each volunteer provided a 5 g fresh stool sample)and put into 200 mL of PBS buffer (1 L of sterilized water, 0.24 g of potassium dihydrogen phosphate, 1.44 g of sodium dihydrogen phosphate, 8 g of sodium chloride, and 0.2 g of potassium chloride, stored at room temperature) and shaken well. The fecal pellets were filtered through four layers of gauze and centrifuged at 8000 rpm for 1 min at room temperature to remove the small fecal pellets, and the mixing bacterial suspension obtained was used for subsequent inoculation; the whole operation was performed under aseptic conditions.

#### 2.3.2. Simulation of In Vitro Fermentation

The in vitro fermentation process was carried out in an intestinal microecological chemotroph simulator system (Minipod 500 mL × 4, T&J Bio-engineering (Shanghai) Co., Ltd., Shanghai, China). The simulation system consisted of four glass tanks for simulating the continuous fermentation process in the distal colon of different DFCs and introducing anaerobic conditions by continuous injection of oxygen-free nitrogen, with precise independent control of culture parameters such as pH, DO, rotational speed, temperature, and micro-recharge rate throughout the process. Dietary fiber and culture medium (peptone 3 g/L, yeast extract 4.5 g/L, bile salt 0.4 g/L, cysteamine hydrochloride 0.8 g/L, heme 0.05 g/L, sodium chloride 4.5 g/L, potassium chloride 2.5 g/L, magnesium chloride hexahydrate 4.5 g/L, calcium chloride hexahydrate 0.2 g/L, potassium dihydrogen phosphate 0.2 g/L, trace element solution 21 mL/L (1 mL/L, Tween 80 1 mL/L)) were placed into the fermenter and marked. After sterilization, all fermenters, connections (after connection), fermentation medium, PBS buffer, and other supplies were sterilized in an autoclave (GR85DP, Zhiwei (Xiamen) Instruments Co., Ltd., Xiamen, China) at 121 °C for 20 min. After sterilization, all parts of the fermenters were connected, the fecal dilutions were inoculated into the fermenters with a sterile syringe, the magnetic stirrer was turned on, the pH electrode was inserted, fermentation was started, and samples were taken for testing.

#### 2.3.3. Extraction of Fermentation Broth DNA

Bacterial DNA was extracted from fecal samples using a fecal genomic DNA extraction kit (Solarbio, Beijing, China). Nucleic acids were obtained from 200 mg of fecal samples and eluted in 90 µL of elution buffer. The concentration of DNA was measured using an infinite M200 pro continuous wavelength multifunctional microtiter detector (Tecan, Männedorf, Switzerland). The extracted DNA samples were stored at −80 °C.

#### 2.3.4. Real-Time Fluorescence Quantitative PCR

A total of five bacteria were quantified from each fecal DNA sample by real-time fluorescent qPCR. DNA amplification and detection were performed using a Roche LightCycler 96 real-time fluorescent qPCR instrument (Roche Diagnostics Co., Ltd., Basel, Switzerland) using optical grade 96-well plates. The samples were routinely analyzed using SYBR Green qPCR Master Mix in a total volume of 20 µL. Each reaction consisted of 2 µL template DNA, 7 µL ddH_2_O, 10.0 µL 2 × SYBR qPCR Master Mix (Vazyme Biotechnology Co., Ltd., Nanjing, China), 0.5 µL primer 1, and primer 2 (10 µM), and real-time PCR conditions included an initial denaturation step at 95 °C for 5 min and an amplification step, followed by 40 cycles of denaturation at 95 °C for 30 s, annealing of primers at optimal annealing temperature (Table 1), extension at 72 °C for 30 s, and extension at 72 °C for another 8 min. At the end of the PCR assay, dissociation curve analysis was performed to check for nonspecific products and/or SYBR Green probe contamination. The relative quantification method was used, and the relative expression of the genus is expressed according to the following formula:

(3) $$\mathrm{Relative}\;\mathrm{expression}\;\mathrm{level}\;=\;{}_{2-}\{\left(\mathrm{Ct}\;\mathrm{value}\;\mathrm{of}\;\mathrm{target}\;\mathrm{gene}\;\mathrm{to}\;\mathrm{be}\;\mathrm{tested}-\mathrm{Ct}\;\mathrm{value}\;\mathrm{of}\;\mathrm{internal}\;\mathrm{gene}\;\mathrm{to}\;\mathrm{be}\;\mathrm{tested}\right)-\;\left(\mathrm{Ct}\;\mathrm{value}\;\mathrm{of}\;\mathrm{control}\;\mathrm{target}\;\mathrm{gene}-\mathrm{Ct}\;\mathrm{value}\;\mathrm{of}\;\mathrm{control}\;\mathrm{internal}\;\mathrm{reference}\;\mathrm{gene}\right)\}$$

### 2.4. ^1^H NMR Spectral Analysis

#### 2.4.1. Sample Preparation

The above fermentation broth samples were removed and placed in a refrigerator at −80 °C and thawed at room temperature, and then, 1000 μL of supernatant was placed in a 2 mL centrifuge tube (Hamburg, Germany) and centrifuged at 10,000 rpm for 10 min. A total of 500 µL of PBS/D_2_O buffer (0.1 M, pH 7.4) was added, containing 10% D_2_O (*v*/*v*) and 0.005% TSP (*w*/*v*), vortexed for 15 s, mixed and repeatedly freeze–thawed with liquid nitrogen three times. The mixture was homogenized for 60 s, followed by centrifugation (12,000 rpm, 10 min, 4 °C). Supernatants were removed, and the extraction procedure was repeated. The supernatants from two extractions were collected for centrifugation (12,000 rpm, 15 min, 4 °C). Then, 550 µL of the resulting supernatant was transferred into a 5 mm NMR tube for further analysis.

#### 2.4.2. Acquisition and Processing of ^1^H NMR Data

^1^H NMR spectra of the feces were measured by a Bruker Avance 500 MHz NMR spectrometer (Bruker, Karlsruhe, Germany) at a ^1^H frequency of 500.13 MHz and a temperature of 298 K. Fecal extract samples were recorded using the water pre-saturated standard one-dimensional NOESYPR1D pulse sequence (recycle delay − 90° − t_1_ − 90° − t_m_ − 90° − acquisition) with water suppression to obtain the representative total metabolite compositions. The acquisition parameters were as follows: relaxation delay, 2.0 s; mixing time, 0.1 s; number of scans, 64; spectral size, 65,536 points; spectral width, 10,000 Hz. MestReNova (version 14.0, Mestrelab Research, Santiago de Compostela, Spain) was used to obtain the NMR spectra. All free induction decays (FIDs) from 1D ^1^H NMR were multiplied by a 0.5 Hz exponential line broadening before Fourier transformation. After corrections for phase and baseline distortion, ^1^H NMR spectra were referenced to the TSP signal (δ 0.0), and the spectral regions of 0.00–9.00 ppm were integrated into regions with equal widths of 0.001 ppm. The water signal, dominating the spectrum between 4.70 ppm and 5.10 ppm, was excluded to eliminate the spurious effect of water suppression, and the integration data were normalized for further analysis.

#### 2.4.3. Metabolomics Analysis

Multivariate data analysis was performed using the Metaboanalyst website (www.metaboanalyst.ca. accessed on 10 October 2022). An unsupervised pattern recognition analysis method, principal component analysis (PCA), was used to examine any intrinsic clustering of the data. In addition, heat mapping of correlations between metabolites of different DFCs and characteristic human flora was performed on the Omicstudio website (www.omicstudio.cn. accessed on 20 November 2022). Metabolic pathway enrichment and pathway analysis were performed based on the KEGG pathway database to identify the metabolic pathways of different DFCs. Redundancy analysis was performed by R language to determine the relationship between major differential metabolites and fiber content.

### 2.5. Statistical Analysis

The results are expressed as mean ± standard deviation. All statistical analyses were performed using SPSS 22.0 (SPSS Inc., Chicago, IL, USA). One-way analysis of variance (ANOVA) was used for multiple group comparisons. Differences were considered statistically significant when *p* < 0.01 or *p* < 0.05.

## 3. Results

### 3.1. Nutrient Composition and Fiber Content Analysis

**Figure 1:**
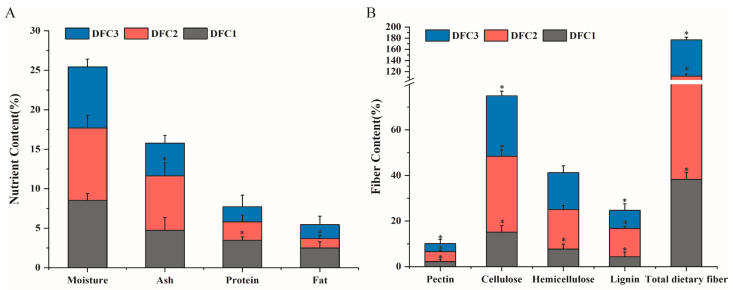
(**A**) Basic nutrient content of different DFCs. (**B**) Various types of fiber content of different DFCs. *: *p* < 0.05, significance of nutrient or fiber content between different DFCs in the same column.

To investigate the beneficial effects of DFCs on human intestinal health mediated by the longevity dietary pattern, we first needed to understand its nutritional composition and fiber content. In this study, nine basic components of water, ash, fat, protein, pectin, cellulose, hemicellulose, cellulose, and total dietary fiber were determined. The analysis results show (Figure 1) that, after extraction and optimization, the pectin, cellulose, hemicellulose, lignin, and total dietary fiber contents of DFC2 were higher than those of DFC1 and DFC3, and impurities, such as protein and fat, were lower in DFC2 and DFC3 than in DFC1. Studies have shown that the maintenance of human intestinal microecological balance is closely related to the regulation of dietary fiber [26,27]. We can tentatively conclude that there is variability in the regulation of gut microbes by DFCs with different fiber contents, so it is necessary to further explore the effects produced by different DFCs on human gut microbes.

### 3.2. Effect of Different DFCs on Characteristic Human Flora after In Vitro Fermentation

**Figure 2:**
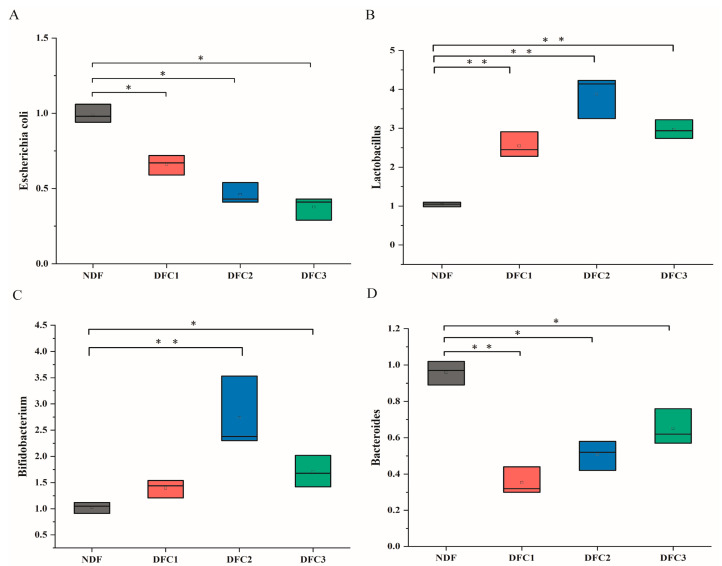
(**A**) Relative expression of *Escherichia coli* after different DFC fermentations. (**B**) Relative expression of *Lactobacillus* after different DFC fermentations. (**C**) Relative expression of *Bifidobacterium* after different DFC fermentations. (**D**) Relative expression of *Bacteroides* after different DFC fermentations. * *p* < 0.05; **, *p* < 0.01, significance relative to the NDF group.

The changes in characteristic human flora of different DFCs after in vitro fermentation are shown in Figure 2. Compared with the NDF group, DFC1 increased the levels of *Lactobacillus* (*p* < 0.01) and inhibited the levels of *Escherichia coli.* and *Bacteroides* (*p* < 0.01) but had little effect on *Bifidobacterium* (*p* > 0.05). DFC2 increased the levels of *Lactobacillus* and *Bifidobacterium* (*p* < 0.01) and inhibited the levels of *Escherichia coli* and *Bacteroides* (*p* < 0.05), whereas the relative expression of *Lactobacillus* and *Bifidobacterium* increased by 271.20% and 166.60%, respectively. DFC3 increased the levels of *Lactobacillus* (*p* < 0.05) and *Bifidobacterium* (*p* < 0.01) and decreased the levels of *Escherichia coli* and *Bacteroides* (*p* < 0.05), whereas the relative expression of *Lactobacillus* and *Bifidobacterium* increased by 184.30% and 66.20%, respectively. Overall, the different DFC interventions increased the levels of *Lactobacillus* and *Bifidobacterium* and suppressed the levels of *Escherichia coli* and *Bacteroides*.

### 3.3. Effect of Different DFCs on Metabolites after In Vitro Fermentation

#### 3.3.1. Identification and Comparison of Metabolites

**Figure 3:**
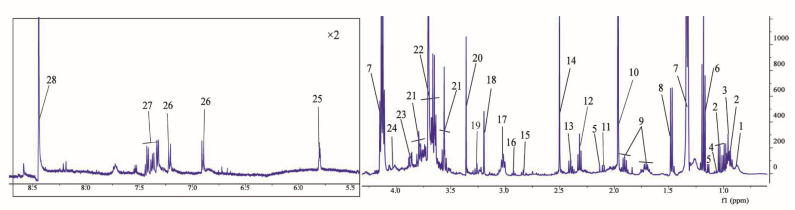
Representative ^1^H NMR metabolic spectrum of DFC after 48 h in vitro fermentation. Note: 1, valeric acid; 2, isoleucine; 3, leucine; 4, valine; 5, propionic acid; 6, ethanol; 7, lactic acid; 8, alanine; 9, lysine; 10, acetic acid; 11, butyric acid; 12, glutamic acid; 13, succinic acid; 14, citric acid; 15, dimethylamine; 16, trimethylamine; 17, isobutyric acid; 18, choline; 19, betaine; 20, methanol; 21, β-glucose; 22, α-glucose; 23, aspartic acid; 24, histidine; 25, uracil; 26, tyrosine; 27, phenylalanine; 28, formic acid.

The representative metabolic ^1^H NMR spectra of DFC samples after 48 h fermentation are shown in Figure 3. The metabolic spectra after fermentation exhibited generally similar chemical shifts, indicating that the DFC samples had similar chemical distributions after in vitro fermentation. The major ^1^H peaks were in the range of 0.5–5.5 ppm, and the fecal referenced signals were mainly composed of glycans (α-glucose and β-glucose), short-chain fatty acids (SCFAs) (acetic acid, butyric acid, and propionic acid), organic acids (lactic acid, succinic acid, and citric acid), biogenic amines (dimethylamine and trimethylamine), amino acids (alanine, aspartic acid, and glutamic acid), and other lipids (choline and betaine).

The metabolites identified in this study were mainly distributed in the range of 0.5–8.5 ppm and included sugars, amino acids, fatty acids, and other metabolites (Figure 3, Table S1). The ^1^H signals in the region around 0.8–2.5 ppm represent amino acids (isoleucine, leucine, valine, alanine, and lysine), SCFAs (acetic acid and butyric acid), and organic acids (lactic acid and succinic acid). The ^1^H peak in the region of 2.5–5.5 ppm indicates the presence of sugars, such as α-glucose and β-glucose. Others include amino acids (aspartic acid and lysine), biogenic amines (trimethylamine), and organic acids (lactic acid). Finally, amino acids such as phenylalanine and tyrosine were found in the region around 5.5–8.5 ppm.

#### 3.3.2. Multivariate Statistical Analysis of Different DFCs on Metabolites after In Vitro Fermentation

**Figure 4:**
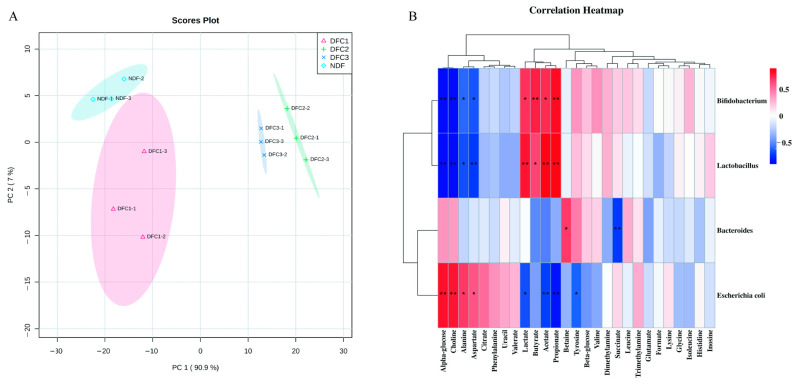
(**A**) Scatter plot of PCA scores (**B**) Heat map of correlation between metabolites and characteristic human flora of different DFCs and NDF. Note: *, *p* < 0.05; **, *p* < 0.01.

To characterize the overall metabolic effects of different DFCs, a multivariate statistical analysis of the ^1^HNMR spectra was performed in this study. The ^1^HNMR spectra were normalized and a PCA principal component analysis was used to explore the metabolite distribution of NDF, DFC1, DFC2, and DF3 (PC1 and PC2 explained 90.9% and 7% of the total variance, respectively), and the PCA score plot (Figure 4A) showed that there was good separation between different DFCs and NDF after in vitro fermentation, indicating that the metabolites after fermentation could be well differentiated by the model. The correlation heat map (Figure 4B) further characterized the correlations between the metabolites of different DFCs and the characteristic human flora, with *Bifidobacterium* and *Lactobacillus* positively correlated with acetic acid, propionic acid, butyric acid, and lactic acid, and negatively correlated with aspartic acid, alanine, choline, and α-glucose; *Bacteroides* positively correlated with betaine and negatively correlated with succinic acid; *Escherichia coli* positively correlated with α-glucose, choline, alanine, and aspartic acid and it was negatively correlated with lactic acid, acetic acid, propionic acid, and tyrosine.

#### 3.3.3. Screening of Differential Metabolites in Fermentation Broth after In Vitro Fermentation with Different DFCs

**Figure 5:**
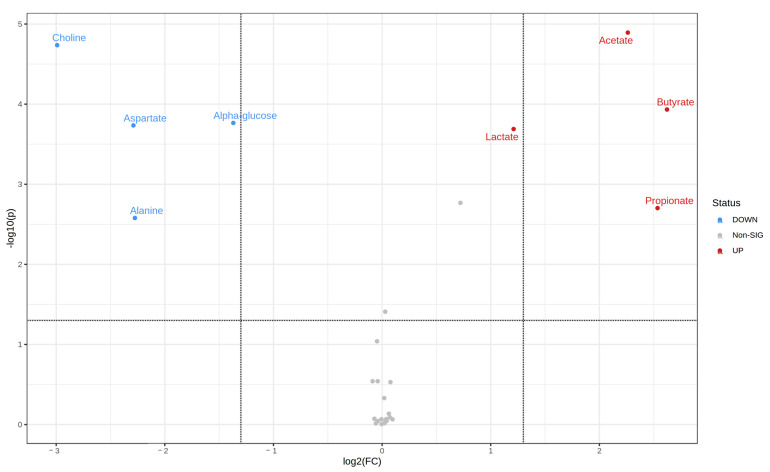
Representative volcano plots of major differential metabolites after DFC fermentation (i.e., volcano plots of DFC2). Note: Metabolites significantly up-regulated in red, metabolites significantly down-regulated in blue, and metabolites with no significant differences in gray.

To screen the significantly different metabolites after in vitro fermentation, this study used volcano plots (Figure 5 and Figure S1) to characterize the range of up- and down-regulation of the main differential metabolites between the different DFC samples and the NDF group, combined with log_2_Fold Change (log_2_FC) values, *p*-values (*p* < 0.05), and VIP values (VIP > 1) (Table 2) to identify the different significantly different metabolite species. The results show that different DFC interventions after fermentation had different effects on different metabolites. Compared with the NDF, first, four metabolites of DFC1 were significantly changed; three metabolites were significantly up-regulated, namely, acetic acid, propionic acid, and succinic acid; and one metabolite was significantly down-regulated, betaine. Second, eight metabolites of DFC2 showed significant changes; four metabolites were significantly up-regulated, namely, acetic acid, propionic acid, butyric acid, and lactic acid, and four metabolites were significantly down-regulated, namely, α-glucose, choline, alanine, and aspartic acid. Third, six metabolites of DFC3 showed significant changes; three metabolites were significantly up-regulated, namely, acetic acid, propionic acid, and lactic acid, and three metabolites were significantly down-regulated, namely, α-glucose, choline, and alanine.

#### 3.3.4. Metabolic Pathway Analysis of Different DFCs for Different Metabolites after In Vitro Fermentation

**Figure 6:**
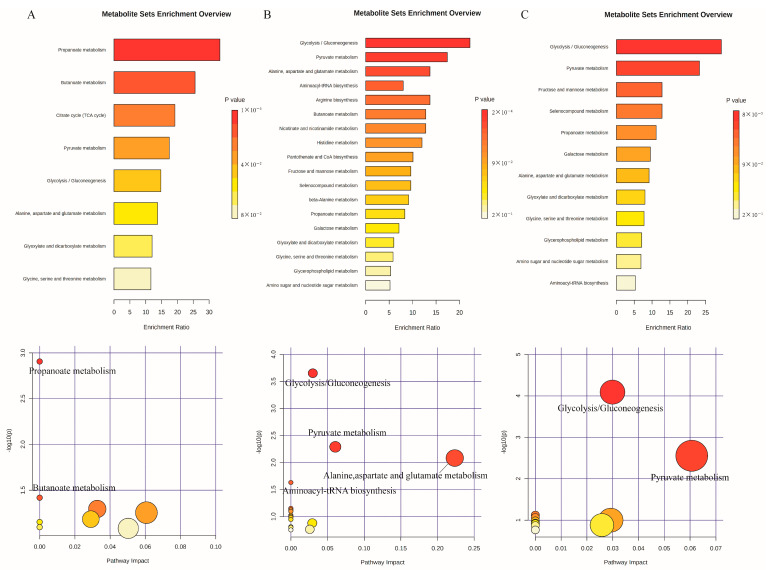
Enrichment analysis and pathway analysis of the main differential metabolites of different DFCs ((**A**–**C**) are DFC1, DFC2, and DFC3 respectively).

To understand whether there are synergistic changes between the main differential metabolites of different DFC and their metabolic pathways, metabolite enrichment and pathway analyses were performed for the main differential metabolites of three groups of DFC samples (4, 8, and 6 species, respectively) (Figure 6A–C). The bubble size represents the effect factor size of the pathway, and the color shade represents the *p*-value size of the enrichment analysis. The results show that two of the four major differential metabolites of DFC1 were significantly altered (*p* < 0.05) in propionate metabolism and butyrate metabolism, respectively. Four of the eight major differential metabolites of DFC2 were significantly altered (*p* < 0.05) in glycolysis/gluconeogenesis; pyruvate metabolism; alanine, aspartate, and glutamate metabolism; and aminoacyl-tRNA biosynthesis, respectively. Two of the six major differential metabolites of DFC3 were significantly altered (*p* < 0.05) in glycolysis/gluconeogenesis and pyruvate metabolism, respectively.

### 3.4. Redundancy Analysis between Fiber Content, Metabolites, and Characteristic Human Flora

**Figure 7:**
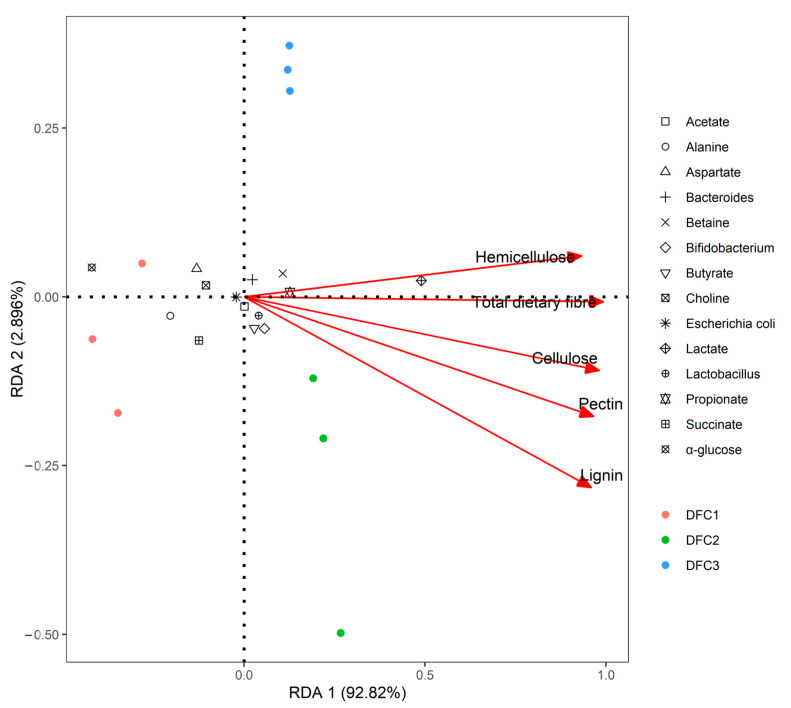
Redundancy analysis between the fiber content of different DFCs and metabolites and gut microbes.

To investigate the relationship between the fiber content of different DFCs and different metabolites and characteristic human flora, a redundancy analysis was performed in this study (Figure 7). The results show that the interval distribution between the fiber content of different DFCs and different metabolites and characteristic human flora was different, and the RDA 1st and 2nd sorting axes explained 92.82% and 2.89% of the percentage changes, respectively; along the 1st sorting axis, from left to right, DFC1, DFC2, and DFC3 showed negative, positive, and positive correlations with the fiber content, respectively. DFC2 was closer to the fiber content than DFC3, indicating that the positive correlation between DFC2 and fiber content was more obvious. The fiber content in order from more to less is DFC2, DFC3, and DFC1, which was consistent with the results of the study in Section 3.1. From the spatial clustering of fiber content with metabolites and characteristic human flora, fiber content was positively correlated with *Lactobacillus*, *Bifidobacterium*, butyric acid, propionic acid, acetic acid, *Bacteroides*, lactic acid, and betaine, and *Escherichia coli*, succinic acid, alanine, choline, aspartic acid, and α-glucose were negatively correlated.

## 4. Discussion

In recent years, many studies have shown the effects of DF on human intestinal health [28,29,30]. Therefore, there is increasing interest and active research on its fermentable fraction in dietary composition. In this study, fresh fecal intestinal microorganisms from people aged 65–75 years were used as fermenting organisms. Different proportions and types of dietary fiber compounds were used as fermentation substrates, and the fermentation simulation system was used to investigate the effect of dietary fiber compounds on the regulation of characteristic human flora and metabolites in the intestine in the longevity diet pattern of Guangxi.

This study found that different dietary fiber compounds had different effects on the characteristic human flora in the intestine. DFC1 increased the level of *Lactobacillus*; DFC2 promoted the growth of *Lactobacillus* and *Bifidobacterium* more significantly than DFC3 (*p* < 0.01). DFC decreased the level of *Escherichia coli* in all three groups. Sun [31] found that the in vitro fermentation of arabinogalactan promoted the proliferation of *Bifidobacterium* and *Brauterium* and inhibited the growth of unclassified *Enterobacteriaceae* and *Citrobacter*. Yang [32] found that pectin increased *Bifidobacterium* by almost 25% after in vitro fermentation compared to the control group. A study comparing commercial dietary fibers (resistant starch, pectin, and polydextrose) with orange albedo found that orange albedo had an optimal stimulation of the growth and metabolism of *Lactobacillus* and *Bifidobacterium* species [33]. In conclusion, the results of this study indicate that that DFCs in the longevity dietary pattern mediated the proliferation of (*Lactobacillus* and *Bifidobacterium*) probiotics and inhibition of (*Escherichia coli*) harmful bacteria, and DFC2 produced more positive effects.

This study found that different DFCs have different major differential metabolites, metabolic pathways, and different roles in the intestinal metabolic environment. After the DFC1 intervention, the differential metabolites were mainly enriched in the propionic acid and butyric acid metabolic pathways, which are cholesterol-lowering, anti-inflammatory, and anti-cancer [34,35], indicating that DFC1 acts in the intestine mainly by regulating short-chain fatty acid metabolism. After the DFC2 intervention, differential metabolites were mainly enriched in glycolysis/gluconeogenesis, pyruvate metabolism, alanine, aspartate and glutamate metabolism, and aminoacyl tRNA biosynthesis, where the glycolysis/gluconeogenesis and pyruvate metabolic pathways play a role in regulating gluconeogenesis and energy metabolism [36,37]. Alanine, aspartate, and glutamate metabolism link lipid and glucose metabolisms through the short-chain fatty acid cycle, and energy production leads to a large accumulation of lipids [38,39,40], suggesting that DFC2 acts in the gut mainly by promoting the sugar and lipid metabolisms and regulating amino acid metabolism. After the DFC3 intervention, differential metabolites are mainly enriched in glycolytic/glycogenic and pyruvate metabolic pathways, suggesting that DFC3 acts in the gut mainly by promoting sugar metabolism. One study found that composite DF from both the Mediterranean and Scandinavian diets produced metabolites during in vitro fermentation, with whole grain DF producing more propionate than refined flour grain DF and fruit and vegetable DF producing more butyrate than grain DF [41]. Boulaka [42] explored the metabolic markers of edible mushrooms containing β-glucan associated with healthy aging in humans and found significant increases in short-chain fatty acids (acetate, propionate, and butyrate), phenylalanine and tyrosine, trimethylamine, and γ-aminobutyric acid (GABA). Bai [43] found that KEGG pathways associated with soluble dietary fiber metabolism and SCFA synthesis include pyruvate metabolism and glycolytic and dicarboxylic acid metabolism. Whereas the effects of different DFCs on metabolites after in vitro fermentation in longevity dietary patterns are less reported, the present study found that different DFCs play a probiotic role in different metabolic pathways in the intestine by producing different metabolites from metabolomics.

The results of the redundancy analysis in this paper show that there were some correlations between different metabolites, characteristic human flora, and fiber content (Figure 7), and it was found that DFC1, DFC2, and DFC3 were negatively, positively, and positively correlated with fiber content, respectively. The positive correlation between DFC2 and fiber content was more obvious. The positive correlation between fiber content and *Lactobacillus*, *Bifidobacterium*, propionic acid, butyric acid, and betaine indicated that DFC2 had more positive effects in promoting the proliferation of the beneficial bacteria of *Lactobacillus* and *Bifidobacterium* and the production of propionic acid, butyric acid, and lactic acid. It also showed a negative correlation with *Escherichia coli*, succinic acid, alanine, choline, aspartic acid, and α-glucose, indicating that DFC2 was more beneficial in inhibiting the proliferation of *Escherichia coli* harmful bacteria and promoting the sugar and lipid metabolisms. Lamichhane [44] found that the in vitro fermentation of polydextrose produced *Bacteroides* positively correlated with propionic acid production. LeBlanc [45] found that *Lactobacillus* correlated with lactic acid, acetic acid, propionic acid, and butyric acid production. In addition, Lu [46] found that the in vitro fermentation of arabinoxylan was positively correlated with *Bifidobacteria* and acetic acid production. While fewer reports have explored the relationship between different metabolites, characteristic human flora, and fiber content of different DFCs in longevity dietary patterns, the results suggest that different DFCs mediated by longevity dietary patterns play a role in improving intestinal health through the production of different characteristic human flora and metabolites, while DFC2 is more significant in promoting sugar and lipid metabolism and the proliferation of beneficial bacteria.

Due to the rich variety of dietary fiber ingredients in the Guangxi longevity dietary pattern, the next step could be to expand the dietary fiber species for further in-depth study. In addition, this study only explored the effects of different DFCs on characteristic human flora after in vitro fermentation, and the relationship between gut microbes and host interactions and their functions is not complete, needing to be further explored by 16srDNA high-throughput sequencing or whole-genome sequencing. In addition, in vitro fermentation models can be used to explore the effects of DFCs on characteristic human flora and also for mechanistic studies and to develop hypotheses in the field of glycolipid and amino acid metabolism, but the adaptation of these changes still needs further validation by in vivo studies due to the general lack of host response and the effects of interactions between enterocytes, bacteria, other microorganisms, immune cells, metabolites from other sources, etc., that often affect metabolite and proliferation outcomes.

## 5. Conclusions

In this study, three different DFC combinations were constructed as a starting point for dietary fiber compounds in the longevity dietary pattern, and the effects of different DFCs on characteristic human flora and metabolites were explored by in vitro fermentation. DFC1 increased the level of *Lactobacillus*; DFC2 promoted the growth of *Lactobacillus* and *Bifidobacterium* more significantly than DFC3, and all three DFCs decreased the level of *Escherichia coli*. The metabolomics analysis found that different DFCs were involved in different metabolic pathways in the intestine and played different roles in regulating sugar, lipid metabolism, and amino acid metabolism. Combined with the redundancy analysis, DFC2 was found to be more significant in promoting the sugar and lipid metabolisms and the proliferation of beneficial bacteria. The above results suggest that different DFCs mediated by longevity dietary patterns have different effects on characteristic human flora and metabolites and that combining cereal, fruit, and vegetable DFCs may be a better regulatory approach to promote the positive development of intestinal health. These findings provide results that support seeking dietary-fiber-matching strategies to improve human intestinal health under the guidance of the longevity dietary pattern and also provide a theoretical guidance for the development of functional dietary fiber products featured in the longevity areas of Guangxi.

## Figures and Tables

**Table 1:** Primer sequences of five species.

Bacteria	Primer Sequence (5′-3′)	Annealing Temperature	References
Total intestinal flora	F:ACTCCTACGGGAGGCAGCAG R:ATTACCGCGGCTGCTGG	64 °C	[21]
*Escherichia coli*	F:GTTAATACCTTTGCTCATTGA R:ACCAGGGTATCTTAATCCTGTT	60 °C	[22]
*Lactobacillus*	F:AGCAGTAGGGAATCTTCCA R:CACCGCTACACATGGAG	60 °C	[23]
*Bifidobacterium*	F:TCGCGTC(C/T)GGTGTGAAAG R:CCACATCCAGC(A/G)TCCAC	58 °C	[24]
*Bacteroides*	F:CTGAACCAGCCAAGTAGCG R:CCGCAAACTTTCACAACTGACTTA	68 °C	[25]

**Table 2:** Summary of the main differential metabolites of different DFCs after fermentation.

Sample	Metabolite	VIP	log_2_(FC)	*p* Value
	Betaine	1.82	−2.8745↓	4.60 × 10^−7^
DFC1	Propionate	1.81	2.5899↑	1.94 × 10^−3^
	Acetate	1.81	2.0674↑	7.80 × 10^−6^
	Succinate	1.8	1.9312↑	3.12 × 10^−7^
	Choline	1.57	−1.743↓	6.52 × 10^−6^
	Acetate	1.56	2.2618↑	1.28 × 10^−5^
	Lactate	1.56	1.2105↑	2.05 × 10^−4^
DFC2	Butyrate	1.55	2.6224↑	1.17 × 10^−4^
	α−glucose	1.55	−1.3696↓	1.72 × 10^−4^
	Aspartate	1.54	−2.2891↓	1.84 × 10^−4^
	Propionate	1.53	2.5354↑	1.99 × 10^−3^
	Alanine	1.49	−2.2754↓	2.64 × 10^−3^
	Choline	1.84	−1.7430↓	6.52 × 10^−6^
	Lactate	1.83	1.0098↑	5.03 × 10^−4^
DFC3	Acetate	1.74	2.1470↑	2.45 × 10^−3^
	Propionate	1.73	2.4202↑	1.60 × 10^−5^
	Alanine	1.62	−2.0183↓	4.97 × 10^−3^
	α−glucose	1.58	−1.0441↓	1.75 × 10^−5^

Note: “↑” indicates an increase in the relative abundance of metabolites after DFC intervention. “↓” indicates that the relative abundance of metabolites decreased after DFC intervention.

## Data Availability

The data in this study are available on request from the author.

## Supplementary Materials

The following supporting information can be downloaded at: https://www.mdpi.com/article/10.3390/nu14235037/s1, Table S1. Assignment of some signals of ^1^H NMR spectra of DFC after 48h of in vitro fermentation as well as their chemical shifts and multiplicity. Figure S1. Volcano plots of the main differential metabolites of different DFCs after fermentation (A, B represent the relative volcano plots between DFC1, DFC3, and NDF, respectively; red are significantly up-regulated metabolites, blue are significantly down-regulated metabolites, and gray are metabolites with no significant difference).
