# Supplementary material for: Effects of Dietary Fiber Compounds on Characteristic Human Flora and Metabolites Mediated by the Longevity Dietary Pattern Analyzed by In Vitro Fermentation
Source: Nutrients. 2022 Nov 26;14(23):5037. doi: 10.3390/nu14235037 (PMC9739654; doi:10.3390/nu14235037)
Supplement: Supplementary file 1 [file nutrients-14-05037-s001.zip › nutrients-2006625-supplementary.pdf]

nutrients

Table S1. Assignment of some signals of ^{1}H NMR spectra of DFC after 48h of in vitro fermentation as well as their chemical shifts and multiplicity.

Number	Metabolites	Groups	^{1} H chemical shifts (ppm) and multiplicity
1	valeric acid	CH3	0.89(t)
2	isoleucine	δCH3;βCH3	1.01(d);0.94(t)
3	leucine	δCH3;βCH3	0.96(d);0.97(d)
4	valine	γCH2	1.04(d)
5	propionic acid	βCH3;αCH2	1.06(t);2.19(q)
6	ethanol	CH2	1.17(t)
7	lactate	βCH3;αCH	1.33(d);4.12(q)
8	alanine	βCH3	1.48(d)
9	lysine	γCH2;βCH2	1.73(m);1.88(m)
10	acetic acid	γCH3	1.92(s)
11	butyric acid	αCH2	2.15(t)
12	glutamate	γCH2	2.36(dt)
13	succinic acid	CH2	2.41(s)
14	citric acid	CH2(1/2)	2.48(s)
15	dimethylamine	CH3	2.70(s)
16	trimethylamine	CH3	2.88(s)
17	isobutyric acid	CH	3.02(m)
18	choline	N(CH3)3	3.20(s)
19	betaine	CH3	3.26(s)
20	methanol	CH3	3.36(s)
21	β-glucose	3-CH;6-CH	3.50(t);3.75(m)
22	α-glucose	2-CH	3.55(dd）
23	aspartic acid	αCH	3.89(dd)
24	histidine	αCH	4.01(m)
25	uracil	5-CH	5.74(d)
26	tyrosine	3,5-CH;2,6-CH	6.91(d);7.20(d)
27	phenylalanine	2,6-CH;4-CH	7.33(d);7.39(m)
28	formic acid	CH	8.45(s)

A

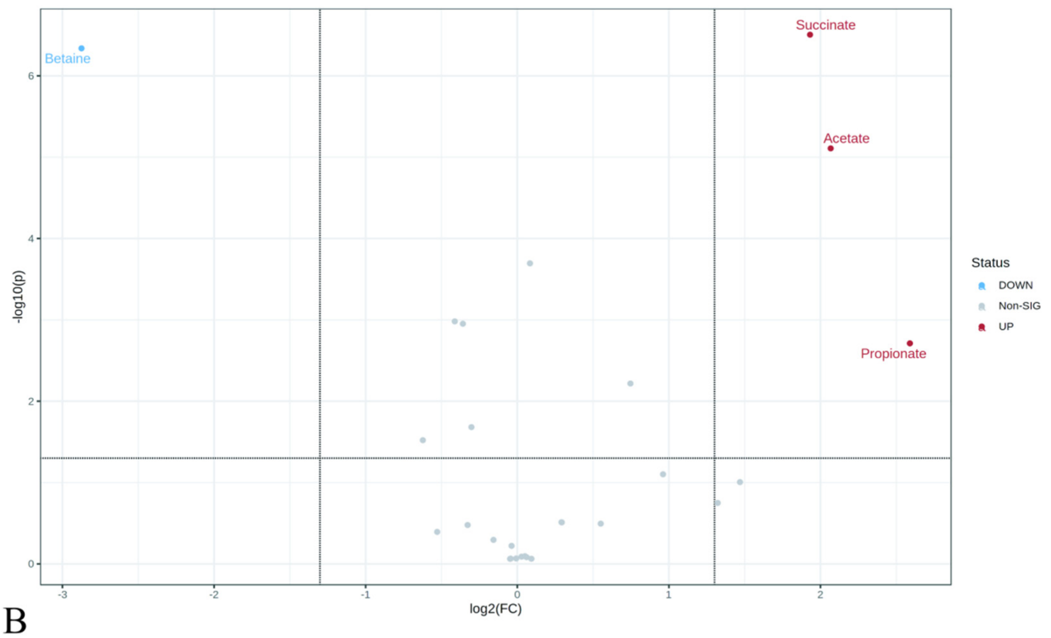

B

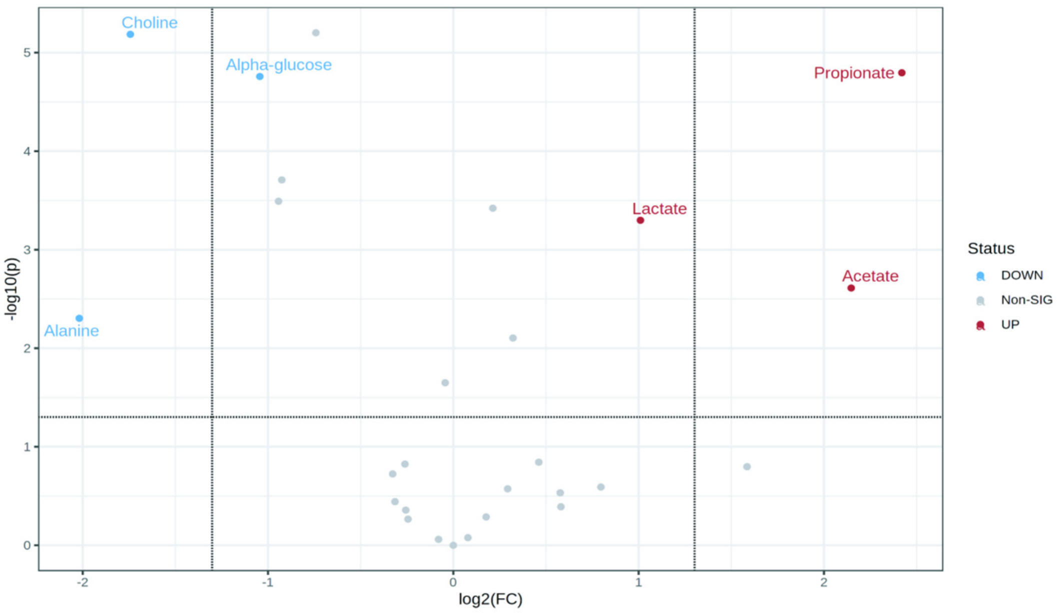

Figure S1. Volcano plots of the main differential metabolites of different DFCs after fermentation (A, B represent the relative volcano plots between DFC1, DFC3, and NDF, respectively; red are significantly up-regulated metabolites, blue are significantly down-regulated metabolites, and gray are metabolites with no significant difference).
